# Head cooling during sleep improves sleep quality in the luteal phase in female university students: A randomized crossover-controlled pilot study

**DOI:** 10.1371/journal.pone.0213706

**Published:** 2019-03-25

**Authors:** Seiji Hamanishi, Eri Eguchi, Tatsuo Ito, Kenjiro Nagaoka, Keiki Ogino

**Affiliations:** 1 Graduate School of Medicine Density and Pharmaceutical Sciences, Okayama University, Okayama, Japan; 2 Department of Nursing, Kansai University of Social Welfare, Ako, Japan; Charité - Universitätsmedizin Berlin, GERMANY

## Abstract

Although selective head-cooling has been reported to decrease scalp and tympanic temperature and improve sleep quality, whether head-cooling during sleep can improve sleep quality in women during the luteal phase has not been elucidated. This randomized, controlled crossover open trial aimed to investigate the effect of head cooling during sleep on sleep quality in women during the luteal phase. Female university students aged 19–25 years with increased daytime sleepiness during the luteal phase were recruited by poster advertisement at their university from May to June 2016 and from May to June 2017. Fourteen women aged 19–22 years participated in this study. The temperature-controllable cooling sheet containing tubes filled with circulating water was used for head-cooling, and the head-cooling and the controlled temperature were set at 25°C and 35°C, respectively. Electroencephalogram data were obtained using a single-channel portable electroencephalogram device. The difference in sleep-related variables and tympanic temperature between head-cooling and control were analyzed using a linear mixed model. The proportion of arousal was lower with head cooling than with the control. In contrast, the proportion of non-REM3 and the delta power were higher with head cooling than with the control. The proportion of non-REM2 and non-REM3 among sleep EEG stages were positively and negatively correlated with the mean tympanic temperature during sleep, respectively. However, arousal and REM were not correlated with tympanic temperature. We considered the reduction of arousal time by head-cooling might be related to scalp temperature rather than tympanic temperature. Further, our results suggested that head-cooling also improved subjective sleep comfort. In conclusion, head-cooling during sleep could improve sleep quality in young women during the luteal phase.

## Introduction

The menstrual cycle is regulated by ovarian hormones including estrogen and progesterone, and many women repeatedly experience physical and psychological changes during menstruation or the luteal phase [1, 2]. Premenstrual syndrome (PMS) refers to the behavioral or social dysfunction due to physical or psychological symptoms during the luteal phase [1, 2]. In particular, the severe psychiatric symptoms of premenstrual dysphoric disorder (PMDD), a severe form of PMS, could impair the social life and human relations of reproductive-age women [3–5]. The sleep-related problem is one of the major premenstrual symptoms. Premenstrual hypersomnia and daytime sleepiness in reproductive-age women have been reported, with their prevalence being higher in patients with PMS or PMDD [4]. Premenstrual hypersomnia and sleepiness are most likely influenced by poor sleep quality [6, 7]. It is suggested that, among the sleep stages determined using electroencephalogram (EEG), rapid eye movement (REM) decreases, whereas arousal and non-REM stage 2 increase in the luteal phase [8–12]. Moreover, previous studies reported that subjective sleep quality deteriorates in the late luteal phase [6, 12, 13]. Changes in sleep quality during the luteal phase may be affected by an increase in body temperature due to increased progesterone [14]. As progesterone inhibits heat loss, the core body temperature increases by 0.3–0.5°C and the temperature rhythm amplitude decreases in the luteal phase [15]. Consequently, the core body temperature remains high during sleep [13–15]. Sleep quality is affected by the core body temperature, with sleep being inhibited unless the body temperature sufficiently decreases [16–18]. Several studies reported that selective head-cooling could decrease scalp and tympanic temperature and improve sleep quality [19–21]. However, to our knowledge, whether head-cooling during sleep can improve sleep quality in women during the luteal phase has not been elucidated. This study aimed to elucidate whether head cooling during sleep can improve sleep quality in women during the luteal phase.

## Materials and methods

### Study design and participants

**Fig 1:**
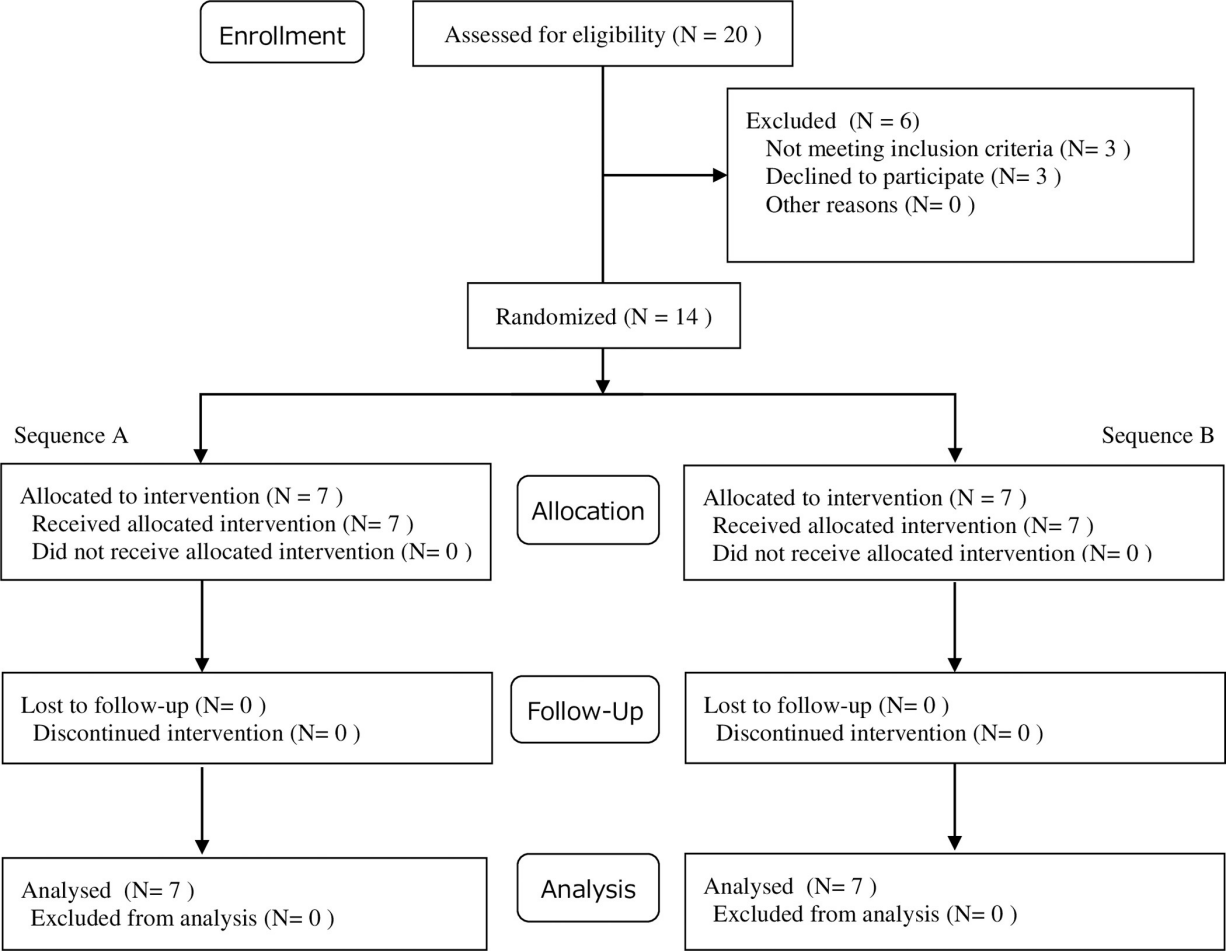
A flowchart of the study design.

We investigated the effect of head cooling on sleep quality in women during the luteal phase in a randomized, controlled, crossover open trial. Female university students aged 19–25 years with increased daytime sleepiness in their luteal phase were recruited by advertisement using posters on the bulletin board at their university in Hyogo, Japan, from May 2 to June 30, 2016, and from May 1 to June 30, 2017. Daytime sleepiness in participants was assessed using the Japanese version of the Epworth sleepiness scale (JESS) [22, 23]. Women with mental illness, gynecological diseases, sleep disorders, or irregular menstruation, as well as pregnant or breastfeeding women, were excluded from this study. The study flowchart is shown in Fig 1. A total of 14 female students participated in this study. All participants were randomly allocated to either sequence A or B using random numbers generated in Excel. The sample size was determined based on the results of the pilot study regarding sleep quality in young women using Sleep Scan (Tanita, Tokyo, Japan). In the pilot study, the mean proportion of the arousal with and without head cooling were 2.96 ± 2.31, 5.01 ± 4.10, respectively. We determined the sample size as fourteen (actual power 81.0%), using the effect size, α error, and power (1-β) at 0.25, 0.05 and 0.8, respectively. The power calculation in this study was performed using G* power [24].

### Study schedule

**Fig 2:**
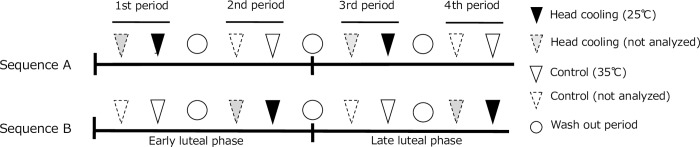
Experimental schedule for the two groups. Black and white triangles indicate head cooling (25°C) and control (35°C), respectively. White circles indicate the one-day washout period. The analyzed data were obtained from the second night each period.

The study schedule is shown in Fig 2. All interventions and examinations were performed from May to June 2016, from September to November 2016, and from September to November 2017. Four intervention periods were set in this study; the first two periods were regarded as the early luteal phase, whereas the other two periods were considered the late luteal phase. Interventions and measurements were performed for two consecutive nights at each period, and we obtained the analyzed data from second night each experimental period. We also inserted one day washout period between each experimental period. The start date of the experiment was determined by increases in participants’ basal body temperature.

### Ethical considerations

Ethical approval was obtained from the Human Ethics Review Committee of Kansai University of Social Welfare (approval number: 27–0310). All participants provided written informed consent before the first examination, and this study conformed to the Declaration of Helsinki guidelines. This study was registered with the University Hospital Medical Information Network-Clinical Trials Registry (UMIN-CTR registry ID: UMIN 000031314) after enrollment of participants had begun. The reason for the delay in registration was that in the Human Ethics Review Committee of Kansai University of Social Welfare, the non-invasion trial was not obliged to register to the clinical trial registration system when we began this trial. The authors confirm that all ongoing and related trials for this intervention are registered.

### Interventions

A temperature-controllable cooling sheet (COCOMIN; Thermic Techno Co., Nagano, Japan), which contained tubes filled with circulating water, was used in this study to cool the participants’ scalps. This cooling sheet was kept at 25°C during the head cooling period and 35°Cduring the non-cooling period. All interventions and examinations were performed in a participant’s bedroom. All participants were asked to go to bed in the same clothes at the same time and to regulate their room temperature (25–28°C), humidity (40–60%), and illumination level (0–5.0 lux) during this study. In addition, participants were asked to refrain from taking a nap and drinking any caffeinated or alcoholic beverages. Any adverse reactions due to head-cooling were not observed in this study.

### Tympanic temperature

Tympanic temperature was measured and recorded every 5 min using an LT-2N-13 tympanic temperature sensor and LT-2 temperature data logger (Gram Corporation, Saitama, Japan). The median temperature was calculated every 30 min to evaluate the effect of head-cooling on tympanic temperature during sleep.

### Sleep EEG

Sleep EEG data were obtained using a single-channel portable EEG device (Sleep scope, Sleep Well Co., Osaka, Japan) [25–28]. According to their manual, an electrode of the device was placed on the middle of the forehead, and another electrode was placed on the left mastoid. The raw EEG data were sent to Sleep Well Co. and analyzed by them, but we did not inform them of our study design and intervention. EEG data were analyzed by visual analysis assisted by the artificial intelligence (AI) system which system accumulated PSG data according to the sleep scoring criteria of the American Academy of Sleep Medicine [29]. EEG data were classified into arousal (Stage W), REM sleep (Stage R), and non-REM sleep. Non-REM sleep was further classified into stage 1 (NREM1), stage 2 (NREM2), or stage 3 (NREM3). Sleep period time (SPT) was calculated by subtracting sleep latency (SL) from time in bed (TIB), and total sleep time (TST) was calculated by subtracting wake after sleep onset (WASO) from SPT. Further, sleep efficiency (SE) was calculated dividing TST by TIB. Delta wave power, which reflects deep sleep, was calculated using a high-pass filtering and a fast Fourier transform algorithm.

### Subjective sleep quality

We evaluated subjective sleep quality, including sleep comfort and sleepiness after waking up, using a self-administered questionnaire. Sleep comfort was assessed using a 0–10 numerical rating scale (NRS), with a lower score indicating more comfortable sleep (0 = best possible sleep, 10 = worst possible sleep) [30]. Subjective sleepiness after waking up in participants was assessed using the Japanese version of the Karolinska sleepiness scale (KSS-J), which is a scale with scores ranging from 1 (extremely alert) to 9 (extremely sleepy) [31].

### Statistical analyses

We used a linear mixed-effect model (restricted maximum likelihood estimation) to examine the effect of head-cooling on sleep-related variables including sleep EEG and tympanic temperature. The model included menstrual phase (Early or Late), period (1–4), and intervention (Cooling or Control) as fixed effects, and participants nested within sequence as a random effect. Bonferroni correction for main effect’s comparison was used to adjust confidence intervals.

The correlations between tympanic temperature and the proportion of each sleep stage were analyzed using Spearman’s rank correlation coefficient. In our study, P-value < 0.05 was considered statistically significant. All data were analyzed using SPSS version 25.0 (IBM, Armonk, NY, USA).

## Results

### Baseline characteristics of study participants and summarization for all measurements

The characteristics of participants and the summarization for all measurements including sleep-related variables and tympanic temperature are shown in Table 1. Participants had a mean age of 20.4 (range, 19–22) years, menstrual cycle length of 28.9 (range, 26–35) days, body mass index (BMI) of 21.1 (range, 20–23) kg/m^2^, and JESS scores of 5.93 (range, 2–10) in the follicular phase, 15.1 (range, 9–21) in the luteal phase, and 12.1 (range, 7–20) during menstruation. None of the participants were smokers or had been previously, drank alcohol more than once per week, or regularly exercised for more than 30 min per week. All measurements are presented as the mean value and standard deviation among four periods. There were no differences in all variables between sequence A and B, except TIB, SPT and, TST.

**Table 1:** Baseline characteristics of study participants and summarization for measurements.

	All (n = 14)	Sequence A (n = 7)	Sequence B (n = 7)	P-value
Age	20.36 ± 1.08	20.43 ± 1.40	20.14 ± 1.07	0.36
Menstrual cycle	28.86 ± 2.21	29.29 ± 2.21	28.43 ± 2.30	0.57
Body Mass Index	21.11 ± 1.20	20.79 ± 1.15	21.43 ± 1.24	0.38
Daytime sleepiness				
Follicular phase	5.93 ± 3.08	5.71 ± 3.45	6.14 ± 2.91	0.81
Luteal phase	15.14 ± 3.03	14.29 ± 1.80	16.00 ± 3.87	0.21
Menstruation	12.07 ± 4.45	11.00 ± 5.54	13.14 ± 3.87	0.21
Sleep stage				
Arousal (%)	4.87 ± 2.43	5.24 ± 2.10	4.49 ± 2.84	0.32
REM (%)	21.99 ± 3.93	21.58 ± 5.43	22.40 ± 1.89	0.38
non-REM1 (%)	8.30 ± 2.58	8.04 ± 1.91	8.56 ± 3.26	1.00
non-REM2 (%)	52.66 ± 9.43	53.56 ± 5.44	51.77 ± 12.70	0.90
non-REM3 (%)	12.17 ± 8.17	11.56 ± 3.95	12.78 ± 11.33	1.00
Time in bed (min)	373.31 ± 45.71	397.77 ± 44.14	348.86 ± 34.40	**0.02**
Sleep period time (min)	348.54 ± 34.16	369.57 ± 21.57	327.50 ± 32.10	**0.01**
Total sleep time (min)	331.42 ± 33.87	350.07 ± 22.22	312.77 ± 34.35	**0.04**
Sleep latency (min)	22.49 ± 21.87	25.84 ± 26.13	19.14 ± 18.11	0.81
Wake after sleep onset (min)	17.12 ± 13.10	13.52 ± 6.76	20.71W ± 16.63	0.16
Sleep efficiency (%)	89.19 ± 6.32	88.65 ± 5.43	89.73 ± 7.52	0.46
Delta power (μV^2^/min)	2732.98 ± 899.97	2768.43 ± 544.40	2697.54 ± 1206.48	0.90
Subjective sleep index				
Sleep comfort	6.02 ± 0.65	6.00 ± 0.69	6.04 ± 0.67	1.00
Sleepiness after waking	4.88 ± 0.78	4.82 ± 0.35	4.93 ± 1.09	0.71
Tympanic temperature	35.94 ± 0.22	35.95 ± 0.18	35.94 ± 0.27	0.90

All data are presented as the mean ± SD (min—max). P < 0.05 indicates significant difference between sequence A and B in Mann-Whitney U test.

### Effect of head cooling on sleep related variables and tympanic temperature during sleep time

The intervention effects of head cooling on the sleep-related variables and tympanic temperature during sleep are shown in Table 2. Least-squares (LS) means of the intervention effect in our model are presented as the estimated means. The proportion of arousal (P < 0.01) among sleep stages with head cooling was significantly lower than those without head cooling, while the proportion of non-REM 3 (P = 0.01) and the delta power value (P = 0.03) with head cooling were higher than control. There were also significant differences in the score for sleep comfort (P < 0.01), sleepiness after waking up (P = 0.02), and tympanic temperature (P < 0.01). There were also significant differences in the sleepiness (P < 0.01) between menstrual phase (Early or Late). Further, there were significant differences in the interaction effect (Intervention * Period) in TIB (P = 0.04) and TST (P = 0.03).

**Table 2:** Intervention effect of head cooling on the sleep-related variables and the tympanic temperature.

	Estimated mean		Intervention		Period		Phase		Intervention * Period		Intervention * Phase	
	Cooling	Control	SE	P value	SE	P value	SE	P value	SE	P value	SE	P value
Sleep stage												
Arousal (%)	3.70	6.03	0.66	**<0.01**	0.66	0.80	0.66	0.51	1.49	0.58	0.94	0.66
REM (%)	22.82	21.16	0.98	0.10	0.98	0.64	0.98	0.99	2.38	0.71	1.38	0.72
non-REM1 (%)	8.11	8.49	0.97	0.70	0.97	0.73	0.97	0.42	2.17	0.84	2.29	0.80
non-REM2 (%)	51.13	54.20	1.85	0.11	1.85	0.63	1.85	0.82	5.54	0.74	2.61	0.07
non-REM3 (%)	14.22	10.13	1.38	**<0.01**	1.38	0.53	1.38	0.58	4.74	0.79	1.95	**0.04**
Time in bed (min)	370.12	376.50	10.07	0.53	10.07	0.28	10.07	0.64	23.43	**0.04**	14.24	0.49
Sleep period time (min)	343.54	353.54	9.34	0.29	9.34	0.18	9.34	0.67	19.76	0.06	13.21	0.81
Total sleep time (min)	330.98	331.86	8.95	0.92	8.95	0.17	8.95	0.53	20.12	0.09	12.66	0.99
Sleep latency (min)	24.52	20.46	6.07	0.51	6.07	0.93	6.07	0.19	13.46	0.59	8.58	0.13
Wake after sleep onset (min)	17.54	16.70	3.05	0.78	3.05	0.54	3.05	0.98	5.11	0.11	4.31	0.17
Sleep efficiency (%)	89.81	88.57	1.74	0.48	1.74	0.74	1.74	0.20	3.91	0.76	2.46	0.27
Delta power (μV^2^/min)	2969.39	2496.58	212.19	**0.03**	212.19	0.73	212.19	0.75	543.42	0.89	300.08	0.94
Subjective sleep index												
Sleep comfort	5.46	6.57	0.28	**<0.01**	0.28	0.52	0.28	0.37	0.59	0.95	0.39	0.90
Sleepiness after waking	5.25	4.50	0.31	**0.02**	0.31	0.42	0.31	**<0.01**	0.68	0.97	0.44	0.91
Tympanic temperature	35.83	36.06	0.07	**<0.01**	0.07	0.22	0.07	0.68	0.14	0.89	0.10	0.38

All variables were analyzed by a linear mixed model, and Least-squares (LS) means of intervention effect are presented as the estimated means. P value < 0.05 are indicated in bold digits.

### Analysis of correlation between tympanic temperature and sleep stages on EEG

The correlations between mean tympanic temperature and each sleep stage are shown in Table 3. The mean tympanic temperature during sleep was positively and negatively correlated with non-REM stage 2 (P < 0.01) and non-REM stage 3 (P < 0.01) respectively.

**Table 3:** Spearman’s rank correlations between sleep stages and tympanic temperature.

	Tympanic temperature	
Sleep stage	ρ	p-value
Arousal	-0.03	0.82
REM	-0.24	0.07
non-REM1	0.06	0.68
non-REM2	0.38	**< 0.01**
non-REM3	-0.39	**< 0.01**

P < 0.05 indicates in bold digits.

## Discussion

To our knowledge, the present study is the first to report that head-cooling during sleep can improve sleep quality during the luteal phase in healthy young women with daytime sleepiness or premenstrual hypersomnia. Further, enhancing sleep quality using head-cooling may contribute to improvement in the quality of life in reproductive-age women with daytime sleepiness during the luteal phase.

It is known that body temperature increases by 0.3–0.5°C during the luteal phase, due to progesterone, and that body temperature remains high during sleep [15]. Our results indicated that head-cooling during sleep could decrease the tympanic temperature by approximately 0.2°C during the luteal phase. Previous studies reported that head-cooling under hot conditions decreases the scalp and tympanic temperature during sleep, but not the core body and distal skin temperature [19]. Blood cooled via sweat evaporation in the scalp and forehead flows into the skull via the emissary veins and angular veins, and decreases the brain temperature [32 33]. We considered that the mechanism of selective brain cooling could contribute to the decrease in tympanic temperature by head-cooling.

Arousal during sleep in the luteal phase increases compared with that in the follicular phase [10, 11]. Our results suggested that head-cooling is effective in decreasing the WASO of women in the luteal phase. However, there was no significant correlation between tympanic temperature and the proportion of arousal stage (Stage W), possibly indicating that their WASO in the luteal phase was not affected by the tympanic temperature during sleep. We speculated that the decrease in temperature and bed climate humidity due to head-cooling could reduce their WASO. A bed climate temperature of 32–34°C and relative humidity of 40–60% have been reported to be the comfort bed climate range [34]. The forehead and occipital region are known to be areas sensitive to temperature changes [35]. As head cooling decreases the scalp temperature and perspiration [19], we speculated that the humidity at the contact area between the occipital region and pillow also decreased. We considered that the decrease in temperature and humidity by head-cooling could also contribute to the improvement in subjective comfort in women during the luteal phase.

The delta wave, which is a high-amplitude slow wave with a frequency of ≥ 0.5–2.0 Hz, is a deep sleep parameter, and non-REM stage 3 (SWS) is determined by the presence of ≥ 20% delta waves [3]. SWS is known to be inhibited by thermal stimulation [36]. Moreover, SWS decreases in the luteal phase, while basal body temperature increases, compared with that in the follicular phase [8, 12]. In this study, the proportion of SWS was higher with head-cooling than with control in the luteal phase. Further, there was a significant negative correlation between tympanic temperature and the proportion of SWS. We speculated that the decrease in SWS in the luteal phase was affected by the increase in basal body temperature and that the decrease in tympanic temperature due to head-cooling contributes to the increase in SWS.

Non-REM stage 2 pertains to the sleep stage at which 12–14 Hz sleep spindles are observed [3]. Since allopregnanolone synthesized from progesterone increases sleep spindles, non-REM stage 2 increases in the luteal phase compared with that in the follicular phase [11, 37–39]. In the present study, there was a significant positive correlation between non-REM stage 2 and tympanic temperature during sleep. However, there was no significant difference in non-REM stage 2 between head-cooling and control. We speculated that the proportion of non-REM 2 was affected by the amount of progesterone which increases body temperature of women during the luteal phase but not by head-cooling sufficiently.

Sleeping efficiency (SE), which is calculated by the proportion of TST relative to TIB, is an essential variable used to assess the sleep quality. In our study, head-cooling did not influence the SE of the participants. The previous study with insomnia patients reported that sleep efficiency was not changed by forehead-cooling (14–16°C), while sleep latency was shortened [40]. Since head-cooling did not influence the SE of not only premenstrual hypersomnia but also insomnia, we considered that SE may not be affected by head-cooling during sleep, strongly. In addition, SE in healthy young women has been reported to be stable throughout the menstrual cycle [41–42]. Our results supported the results of previous studies. Although the effects of ovarian hormones on sleep architecture have not been elucidated sufficiently, we considered that the SE in young women is not severely affected by the ovarian hormones including progesterone. Since premenstrual symptoms tend to appear just before menstruation onset, we speculated that the sleep quality also worsens in the late luteal phase rather than the early luteal phase. However, there also were no significant differences in the sleep EEG variables between the early and late luteal phase in our study. The previous studies indicated that the prevalence of PMS increases in women older than 30 years, and the changes in hormonal dynamics due to aging may be related to the severity of premenstrual symptoms [43]. We considered that the bias of the age group (19–22 years) in our participants may have an influence on our results.

Menstrual-related hypersomnia is one of the typical menstrual symptoms [6,9,11,13]. However, there were no significant differences in TIB, SPT, TST and WASO depending on the intervention temperature, experiment period, and menstrual phase. During the experiment period, the participants were asked to go to bed at the same hour, and they woke up to go to university at the same hour. The controlled sleep duration may have affected our results. If the subjects wake up themselves without using an alarm, the different effects could be observed. However, TIB and TSP had significant interaction effects (Intervention * Period). These interaction effects might have meant that the effect of intervention depends upon the period. The short washout period might also have affected results.

Previous studies reported that sleep deprivation leads to psychiatric symptoms, including irritation, depression and increased appetite [44–46]. As they are representative premenstrual symptoms, an improvement in the sleep quality may also contribute to an improvement in other premenstrual symptoms. Future studies should focus on elucidating the effect of continuous head-cooling on sleepiness or other premenstrual symptoms.

There was a significant difference in sleepiness assessed by KSS-J between head cooling and control. Therefore, we considered that enhancing sleep quality by head-cooling could improve daytime sleepiness during the luteal phase. However, our results should be interpreted with caution, since participants were not blinded and their sleepiness were evaluated using self-administered questionnaires. Our results also suggested that sleepiness became stronger in the late luteal phase compared to the early luteal phase. Since allopregnanolone, an allosteric modulator of γ-aminobutyric acid receptor, has a similar effect to benzodiazepine, the increase in allopregnanolone level in the late luteal phase could promote daytime sleepiness. Therefore, an improvement in sleep quality only may not sufficiently decrease daytime sleepiness in the luteal phase.

This study has several strengths. First, to our knowledge, this randomized, controlled, crossover open trial is the first study to show the positive effects of head cooling on sleep quality in young women. As there are individual differences in the severity of premenstrual symptoms, the crossover design could aid in reducing the effect of individual factors on our study results. Second, we consider that head cooling, being safe and straightforward, could help improve the quality of life in many reproductive-age women with premenstrual sleepiness or hypersomnia. Third, as the intervention effect on sleep-related variables was analyzed using a linear mixed model which was taken into consideration the menstrual phase, our results could have been decreased the effect of menstrual phase.

However, this study also has some limitations. First, participants themselves applied for the study in response to poster advertisement, and they may have been more health-oriented than the general population. Also, participants were not blinded in this study. Since the participants' age range was also limited to 19–22 years old, the application of our findings to other ages is limited. Further, since the sample size in our pilot study was not large, small differences might not have detected accurately. Therefore, our results should be considered for these effects. However, future studies that determined the sample size based on our results may be able to verify the validity of our study. The interventions were not performed in an environmentally controlled chamber, and all devices were operated by participants. In order to minimize these effects on our results, we requested the participants to maintain similar room temperature, humidity, and illumination and to use the same nightclothes and bed linens throughout the experimental period. Further, we explained in detail the appropriate usage of the devices to the participants, and the participants adequately practiced repeatedly before the study began. Also, the cooling device and some probes placed on the participants might influence their sleep quality. However, the sound levels generated from the cooling device under the 24.6–26.1 dB condition were 29.4–33.2 dB (25°C) and 29.1–32.8 dB (35°C). Finally, as all interventions and measurements were performed in the same menstrual cycle, the washout period between the intervention periods was set to only one day in this study. Since a short washout period may affect our results, interventions and measurements were performed for two consecutive nights at each period. Further, in consideration of the first night effect, we analyzed the data obtained from the second night.

## Conclusion

Our study showed that head-cooling during sleep decreased the tympanic temperature and improved the part of sleep-related variables including sleep EEG. Our results may support the future study to examine whether head-cooling during sleep may be an effective strategy to improve sleep quality in the luteal phase in reproductive women.

## Supporting information

S1 Checklist CONSORT checklist. (DOC)

S1 Protocol Study protocol. (PDF)

S1 Fig Changes in tympanic temperature during sleep. (TIF)

S2 Fig LS mean plots by each period. (XLSX)
