# Supplementary material for: Head cooling during sleep improves sleep quality in the luteal phase in female university students: A randomized crossover-controlled pilot study
Source: PLoS One. 2019 Mar 25;14(3):e0213706. doi: 10.1371/journal.pone.0213706 (PMC6433270; doi:10.1371/journal.pone.0213706)
Supplement: S1 Protocol — (PDF) [file pone.0213706.s002.pdf]

The main points of clinical study protocol (Translation from Japanese to English)

Title of Study
Head cooling during sleep improves sleep quality in the luteal phase in female university students: A randomized crossover-controlled trial

Research contact person: Seiji Hamanishi
Organization: Kansai University of social welfare
Division: Nursing faculty
Address: 380-3, Shinden, Akoshi, Hyogo
Tel: 0791-46-2545
Email: hamanishi@kusw.ac.jp

Research Institute: Kansai University of Social Welfare
Ethical Review Committee: The ethical committee at Kansai University of Social Welfare.
Approved Date: 22, March, 2016.

Synopsis

Study Protocol and Ethical Consideration

Study objective	To examine the effect of head cooling on sleep quality in the luteal phased women.
Study Design	Randomized controlled crossover trial
Participants	1. Female university students aged 19-25 years old.<br>2. Women with daytime sleepiness in the luteal phase.<br>3. Women who joined in the explanation session and gave written informed consent.
Target sample size	14
Exclusion criteria	1. Patient with mental disease<br>2. Patient with gynecological disease<br>3. Patient with sleep disorder<br>4. Patient with other severe disease<br>5. Pregnant or breast-feeding women<br>6. Average menstrual cycle ≤ 24 days<br>7. Average menstrual cycle ≥ 39 days
Groups	Participants are randomly allocated to two groups (Group A, B).
Intervention	Two nights interventions are conducted in the following order. There are wash-out periods (one day) between each intervention period.<br>Group A 1:cooling 2:control 3:cooling 4:control<br>Group B 1:control 2:cooling 3:control 4:cooling<br>Cooling temperature is set at 25 degrees Celsius.<br>Control temperature is set at 35 degrees Celsius.
Primary outcomes	Sleep electroencephalographic variables
Secondary outcomes	Tympanic temperature and Subjective sleep quality<br>Subjective sleepiness after wake up (KSS-J)<br>Sleep comfort (NRS)
Informed consent	Before participating the study, the potential participants must join study information session with sufficient detail including the purpose, the method and ethical consideration and, voluntarily sign the informed consent format. Further, the participants are guaranteed the right to withdraw their consent after entering the study.
Data Management	The anonymous data with identification number excluding names, addresses or telephone numbers are stored in the locked cabinet for five years after this study finished.
Consultation from participants	If the participants hope to ask any questions or withdraw their consent, they can

	contact the authors by referring the telephone number, e-mail address in the handout.
Publication	The findings of this study will be published in publications or presented at scientific meetings.
Financial Disclosure	This study is supported by JSPS KAKENHI, Grant number 16K21527.
Conflict of Interest	The authors declare that no conflict of interests exists.
Planned follow-up period	2016 April – 2018 March

（様式第２号）

研究計画書

平成　28 年　1 月 14 日

申請者
関西福祉大学看護学部
職　名：助教
氏　名：濵西　誠司

１．研究課題

全身浴および頭部冷罨法における月経前の眠気の改善効果の検証　（頭部冷罨法）

２．研究の概要

（１）研究の背景および目的

月経前に限定して生じる心身の不調を月経前症候群といい、仮眠や眠気は代表的な月経前症状の一つである。日中の強い眠気は日常の活動性や業務効率を低下させるなど生活の質を低下させる要因となるため、眠気を改善するためのセルフケア法の開発は女性の生活の質の改善に寄与することが期待できる。良質な睡眠には深部体温の低下が重要な役割を果たしており、深部体温の低下が阻害されると質の高い睡眠は得られにくくなる。月経前（黄体期）にはプロゲステロンの影響で熱放散が抑制されることで夜間も高体温が維持されるため、このことが月経前に睡眠の質を低下させる一因となっている可能性がある。先行研究において、全身浴 (Silva A, et al, 2013)や頭部冷罨法(Okamoto-Mizuno K, et al, 2003)が睡眠の質を低下させる効果が示唆されているが、月経前の睡眠の質の改善に対しても有用であるかは明らかになっていない。そこで、科研費若手 B (16K21527)の研究課題「全身浴および頭部冷罨法における月経前の眠気の改善効果の検証」のうち、本研究では睡眠中の頭部冷罨法が月経前の女性の睡眠の質に及ぼす影響について検証することを研究目的とする。

（２）研究方法

①研究デザイン

クロスオーバー法による無作為比較対照試験

②研究参加者

研究参加者は黄体期に眠気が強くなる 19-25 歳の女子大学生とする。予備調査結果から研究対象者数を 14 名に設定するが、研究期間中の脱落を考慮して最大 16 名まで募集を行う。研究参加候補者は大学内にポスターを掲示して募集し、研究概要の説明会後に文書を用いて同意書を得た者を研究参加者とする。なお、研究参加者の眠気はエプワース睡眠調査票日本語版（JESS）を用いて、黄体期の得点が卵胞期より 5 点以上高いことを確認する。ただし、精神疾患、婦人科疾患、 睡眠障害、その他重篤な疾患がある者および妊娠中および授乳中の者、月経周期が不規則であったり平均月経周期が 24 日以下または 39 日

以上の者は除外する。

③研究の手順

研究参加者はExcelの乱数機能を用いて無作為にA群（頭部冷却→対照条件）とB群（対照条件→頭部冷却）の 2 群に分ける。日中の眠気や過眠を含む月経前症状は特に黄体期後期に生じやすいことが知られているため、本研究では 2 日ずつ４期の介入期間を設定し、前半の２期間を黄体期前期、後半の２期間を黄体期後期と定義し、各介入期間の間には１日のウォッシュアウト期間を設ける。なお、月経開始日より記録した基礎体温の上昇を確認することで黄体期に移行したとみなし介入を開始する。また、全ての介入と測定は研究参加者の寝室で行うため、就寝時間・寝室環境・寝衣と寝具は一定の範囲に保たれるよう依頼する。また、機器が適切に取り扱うことができるよう、事前に繰り返し説明するとともに練習を行うこととする。

④介入条件

循環式冷却シートココミン（サーミックテクノ）を用いて、就寝から起床まで一定温度を維持して頭部を冷却する。なお、予備調査の結果を踏まえ、頭部冷却条件は25℃、対象条件は35℃に設定した。

⑤評価項目

・鼓膜温の変化:鼓膜温用体温センサーLT-2N-13を用いて測定した体温を５分毎にデータロガーLT-2（いずれもGram　Corporation）に記録する。記録した鼓膜温は３０分毎に平均値を求め、睡眠中の変化を評価する。

・睡眠脳波：１チャンネル睡眠脳波計スリープスコープ（スリープウェル）を用いて睡眠脳波を測定する。単極導出法の活性電極は額の中央（Fz）におき、基準電極は乳様突起におく。睡眠脳波の解析は米国睡眠医学会（AASM）による睡眠および随伴イベントの判定マニュアルに従い、スリープウェルによって行われる。

・主観的睡眠指標：睡眠中の快適性（寝心地）の評価にはNumerical Rating Scale (NRS)を用い、起床後の眠気の評価にはカロリンスカ睡眠調査票日本語版（KSS-J）を用いる。

⑥データの解析

参加者の年齢、平均月経周期、JESSなど研究参加者の基本情報および睡眠脳波、主観的睡眠指標についてはMann-Whittney　Utestを用いて、頭部冷却と対照条件との平均値を比較する。睡眠中の鼓膜温の変化および2条件間の比較には2元配置分散分析を行い、多重比較にはBonferonni法を用いる。また、平均鼓膜温と睡眠脳波との関連には Spearman’s correlations test を用いる。すべての統計解析にはSPSSを用い、有意確率は5%に設定する。

⑦研究期間

研究参加者の募集・実施・データ解析について、承認日～平成30年3月31日を予定している。

３．研究実施にあたっての倫理的配慮

（１）インフォームド・コンセントのための手続き

掲示板に掲示したポスターを見て応募した研究参加候補者を対象に説明会を開催し、口頭と文書で研究の目的・方法・倫理的配慮などに関する説明を行う。本研究への参加は任意であり、研究参加の意思は同意書の提出をもって確認する。また、研究の途中であっても同意撤回書を提出することで同意が撤回可能であることと研究参加の諾否によっていかなる不利益も被らないことを口頭ならびに紙面を用いて説明する。

（２）個人情報の取り扱い

本研究はヘルシンキ宣言ならびに人を対象とする医学系研究に関する倫理指針を遵守して実施する。本研究では個人が特定できないよう得られた全ての情報は ID を用いて管理し、申請者の研究室内の鍵付きキャビネットで管理を行う。研究参加者の秘密保持に十分配慮し、研究結果を公表する際は、氏名、生年月日など研究対象者を特定できる情報を含まないようにする。また、研究目的以外に研究で得られた情報は使用しない。本研究で得られた情報は、研究の中止または研究終了後 5 年が経過した後、電子情報は完全に抹消し、紙媒体はシュレッダーで裁断するなど個人情報に十分注意して廃棄する。

（３）研究対象者に生じる負担とその対処方法

本研究では月経に関する情報を取得するため、女性スタッフを雇用し、実験参加に関わるストレスを軽減できるよう努める。本研究では、基礎体温および睡眠日誌や主観的睡眠評価の記録に約 10 分程度要する。また、脳波計や鼓膜温のプローブを装着して寝ることで拘束感を生じる恐れがある。上記の点については研究参加前に伝えた上で研究に参加してもらうとともに。研究終了時に図書カードを謝礼として支払う。また、希望者には可能な範囲で測定結果を返却するとともに結果説明を行うことを予定している。

（４）研究対象者からの相談方法

研究説明会で配布する資料に申請者の電話番号およびメールアドレスを記載し、いつでも質問・相談ができる旨を伝えておく。

（５）研究成果の公表

本研究成果は医療・公衆衛生・看護系のジャーナルおよび学術集会で発表することを予定している。また、科学研究費助成事業データベース KAKEN でも公表される予定である。

４．研究の資金源

本研究は日本学術振興会科学研究費助成金若手 B（16K21527）の支援を受けて実施される。

５．研究の実施に係る利益相反

本研究の実施にあたり、研究者全員に申請すべき利益相反事項は存在しない。

平成　年　月　日

研究協力者　様

研究調査に関する協力のお願い

月経前に生じる心身の不調を月経前症候群（PMS）といい、日中の眠気や不眠などの睡眠障害は多くの女性にみられる症状です。日中の眠気は仕事や学習の生産性を低下させるだけでなく事故を起こすリスクを高めることから、本研究では月経前の眠気の改善に有用なセルフケア法を明らかにすることで若年女性の生活の質の向上に寄与することを目指しております。

良好な睡眠には夜間の円滑な体温低下が必要ですが、月経前は基礎体温が高くなり体温低下が阻害されることで、寝付きや睡眠の質が阻害されると考えられます。高齢者や不眠症患者を対象とした研究では頭部の冷罨法によって深部体温が低下し、睡眠の質を改善することが報告されていますが、月経前の女性に対する有効性については明らかになっておりません。そこで本研究では、頭部冷罨法による月経前の眠気の改善効果について検討するため、研究調査へのご協力をお願いしたいと考えております。

本研究では月経前の睡眠の質に対する頭部冷罨法の効果について検討するため、特殊な枕を用いて頭部を 25℃に冷却した場合と 35℃に保持した場合の体温変化や睡眠の質を比較します。なお、研究期間には基礎体温の計測、睡眠脳波の測定、睡眠中の鼓膜温の調査用紙の記載などをお願いしたいと思います。なお、各種測定や記録用紙の記載方法などは研究参加に同意いただいた後に改めて詳しくご説明いたします。研究期間は月経前 2 週間程度を予定しています（基礎体温測定および睡眠日誌の記録は 1 か月間）。研究にご協力いただいた方には、些少ではございますが謝礼（図書カードなど）をお支払いするとともにご希望の方には研究結果をご提供させていただきますので、健康管理にご利用ください。

本研究では、月経前になると強い眠気が生じる方にご参加いただきたいと考えております。ただし、妊娠中および授乳中の方、月経周期が不規則な方、ホルモン剤（ピルを含む）や抗うつ剤・抗不安薬・その他脳神経系のお薬を飲まれている方は研究結果に影響を及ぼす可能生があるため参加はご遠慮ください。

本研究成果は国内外のジャーナルへの論文発表および学会発表を予定しています。その際、匿名性の保持のためデータは ID で管理するとともに統計的に処理し、研究のあらゆる段階で個人が特定されないようにします。研究データの入ったＵＳＢメモリ、アンケート用紙・同意書等は鍵のかかる場所に保管し、研究終了後 5 年間保管した後完全に消去いたします。本研究は、関西福祉大学看護学部倫理審査委員会の承認を受けて実施されます。この研究への同意の諾否によって、今後の成績などいかなる不利益を被ることはありません。研究への参加・協力・同意の取り消しなどに関してご質問やご意見がございましたら、ご遠慮なく下記までご連絡ください。

上記内容を充分理解した上でご協力いただける方は、同意書に署名してご提出くださいますよう、お願い申し上げます。

研究実施者：濱西　誠司（関西福祉大学看護学部　助教）

連絡先：兵庫県赤穂市新田 380－3

TEL:0791-46-2545

E-mail:hamanishi@kusw.ac.jp

（研究者用）

関西福祉大学　看護学部
研究実施者　　宛

同　意　書

私は、「全身浴および頭部冷罨法における月経前の眠気の改善効果の検証　（頭部冷罨法）」の研究概要について十分に説明を受けました。つきましては、理解した以下の項目について、その内容に「レ」を入れて、この研究に協力することを同意します。

- □ 研究の目的と意義
- □ 研究方法
- □ 研究への参加協力の自由意志と拒否権
- □ プライバシー及び個人情報の保護
- □ 研究結果の公表方法
- □ 研究に関する質問や意見の連絡方法

＊同意書は二部作成いたします。一部は研究責任者にご提出いただき、もう一部は研究協力者が研究終了までお持ちいただきますようお願い申し上げげます。

平成　　　年　　　月　　　日

研究参加者（自署）＿＿＿＿＿＿＿＿＿＿＿＿＿＿.

説明者＿＿＿＿＿＿＿＿＿＿＿＿＿＿＿＿＿＿.

（研究協力者控え）

関西福祉大学　看護学部
研究実施者　　宛

同　意　書

私は、「全身浴および頭部冷罨法における月経前の眠気の改善効果の検証　（頭部冷罨法）」の研究概要について十分に説明を受けました。つきましては、理解した以下の項目について、その内容に「レ」を入れて、この研究に協力することを同意します。

□ 研究の目的と意義
□ 研究方法
□ 研究への参加協力の自由意志と拒否権
□ プライバシー及び個人情報の保護
□ 研究結果の公表方法
□ 研究に関する質問や意見の連絡方法

＊同意書は二部作成いたします。一部は研究責任者にご提出いただき、もう一部は研究協力者が研究終了までお持ちいただきますようお願い申し上げます。

平成　　　年　　　月　　　日

研究参加者（自署）＿＿＿＿＿＿＿＿＿＿＿＿＿＿.

説明者＿＿＿＿＿＿＿＿＿＿＿＿＿＿＿＿＿＿

（研究者用）

関西福祉大学　看護学部
研究実施者　　宛

同意取消書

私は、「全身浴および頭部冷罨法における月経前の眠気の改善効果の検証　（頭部冷罨法）」に関する研究への参加・協力について、その同意を取り消します。

平成　　　年　　　月　　　日

研究参加者（自署）＿＿＿＿＿＿＿＿＿＿＿＿＿＿＿＿＿.

研究実施者＿＿＿＿＿＿＿＿＿＿＿＿＿＿＿＿＿＿＿＿＿

（研究協力者用控え）

関西福祉大学　看護学部
研究実施者　宛

同意取消書

私は、「全身浴および頭部冷罨法における月経前の眠気の改善効果の検証　（頭部冷罨法）」に関する研究への参加・協力について、その同意を取り消します。

平成　　年　　月　　日

研究参加者（自署）＿＿＿＿＿＿＿＿＿＿＿＿＿＿.

研究実施者＿＿＿＿＿＿＿＿＿＿＿＿＿＿＿＿＿

関福大看発第 27-0310 号

様式第４号

倫 理 審 査 結 果 通 知 書 （再）

平成 28 年 3 月 22 日

濱西 誠司 殿

関西福祉大学
学 長　加藤 明

研究課題名：全身浴および頭部冷罨法による月経前の眠気の改善効果の検証

上記課題の実施計画について、平成 28 年 2 月 19 日に看護学部倫理審査部会で再審査し、審査結果の答申を受け、次のとおり決定したので、関西福祉大学倫理審査委員会規程第９条にもとづき通知します。

記

＜判定結果＞

ア．非該当
イ．承認
ウ．不承認
エ．条件付承認

以上
