# Supplementary figures and images for: Head cooling during sleep improves sleep quality in the luteal phase in female university students: A randomized crossover-controlled pilot study
Source: PLoS One. 2019 Mar 25;14(3):e0213706. doi: 10.1371/journal.pone.0213706 (PMC6433270; doi:10.1371/journal.pone.0213706)

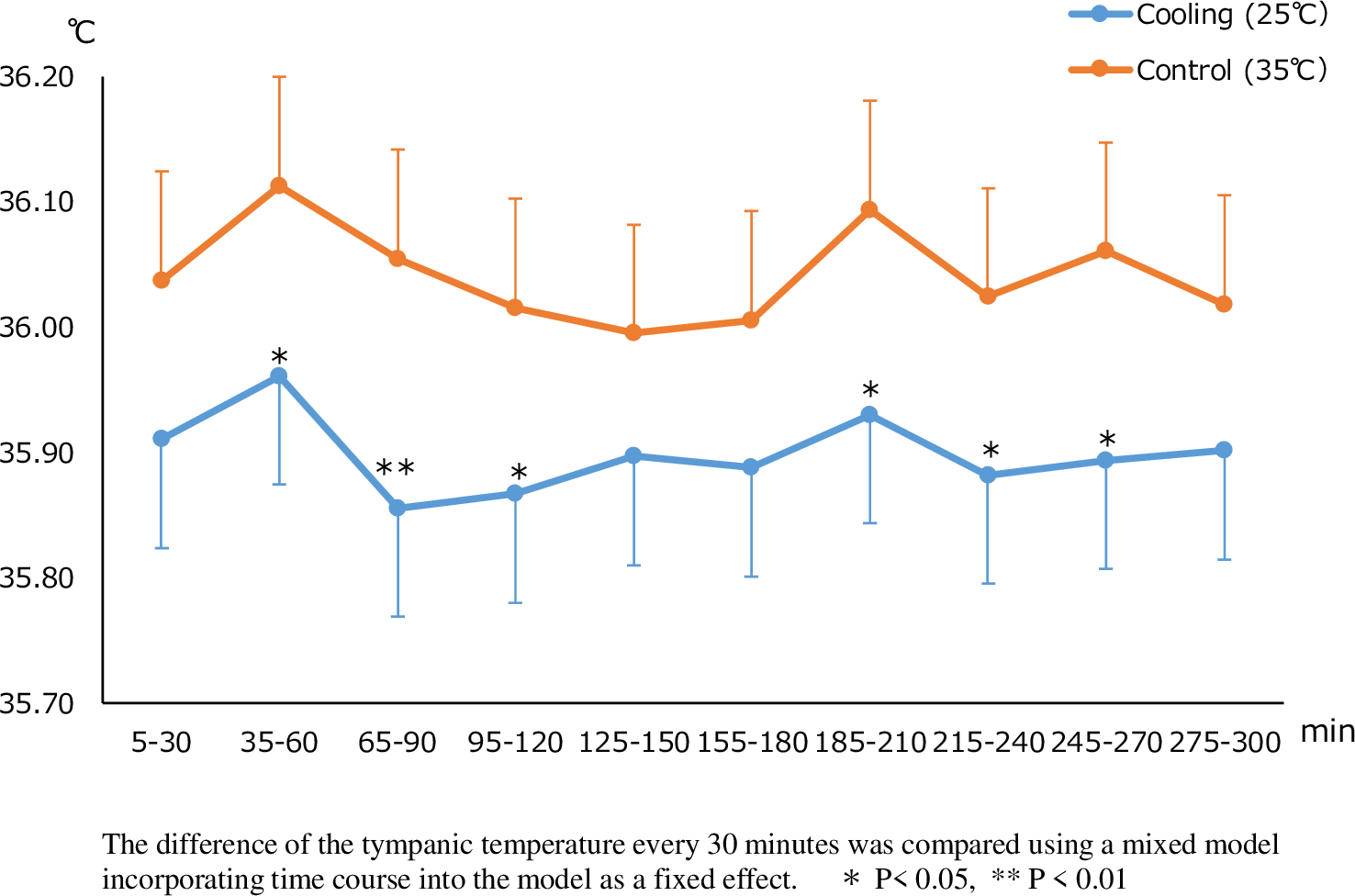

Supplement: S1 Fig — (TIF) [file pone.0213706.s003.tif]
